# Airway epithelial dysfunction in asthma pathogenesis: epigenetic mechanisms, inflammatory crosstalk, and therapeutic opportunities

**DOI:** 10.3389/falgy.2026.1839974

**Published:** 2026-05-22

**Authors:** Bingxue Zhang, Guihua Song, Mengmeng Sun, Yan Zhang, Mingyue Ren

**Affiliations:** 1 Department of Pediatrics, First Affiliated Hospital, Henan University of Chinese Medicine, Zhengzhou, Henan, China; 2 School of Pediatrics, Henan University of Chinese Medicine, Zhengzhou, Henan, China

**Keywords:** airway epithelial barrier, asthma, biologics, DNA methylation, epigenetic memory, epithelial remodeling, trained immunity, type 2 inflammation

## Abstract

Asthma affects over 260 million people worldwide and remains incompletely explained by the traditional T cell-centric immunological model, which offers incomplete mechanistic explanations for disease chronicity, recurrence during clinical remission, and the poor treatment response observed in T2-low phenotypes. Emerging evidence positions the airway epithelium as a central organizer of asthma pathogenesis rather than a passive barrier. This review proposes a unifying framework in which airway epithelial dysfunction and epigenetic memory drive the persistent and relapse-prone nature of asthmatic airways. We first examine how structural barrier defects—including tight junction dysfunction mediated by claudin-18 and E-cadherin loss—initiate and amplify type 2 inflammation through alarmin release (TSLP, IL-33, IL-25) and ILC2 activation. We then review how environmental exposures and inflammatory signals, particularly IL-13, induce durable epigenetic reprogramming of airway epithelial cells through DNA methylation, histone modifications, and non-coding RNAs, establishing molecular imprints that persist beyond the resolution of acute inflammation. Special attention is given to basal progenitor cells as repositories of allergic epigenetic memory, and to the concept of trained innate immunity as a mechanism underlying chronic airway hyperresponsiveness. We further contrast the epigenetic landscapes of T2-high and T2-low asthma, identifying the latter as a critical unmet need for biomarker and therapeutic development. Finally, we discuss translational opportunities, including HDAC inhibitors, miRNA-based therapies, and the potential of anti-alarmin biologics (tezepelumab, itepekimab) and downstream cytokine receptor antagonists (dupilumab) to partially restore epithelial function and progenitor states. We acknowledge that, given current data availability, this review is weighted toward T2-high (eosinophilic) endotypes; mechanistic characterization of T2-low asthma remains an important area for future investigation. This framework reconceptualizes asthma not only as a disorder of dysregulated immunity, but as a disease of maladaptively reprogrammed barrier tissue, with important implications for disease prevention, endotype-specific treatment, and the goal of achieving true biological remission.

## 1 Part I: Introduction

### 1.1 Asthma beyond the T-cell-centric model

Asthma affects approximately 260 million individuals worldwide, representing one of the most common chronic respiratory diseases (1). Although age-standardized prevalence rates have declined over the past three decades, the absolute number of affected individuals continues to rise, driven by population growth and increased exposure to modifiable risk factors—including obesity, smoking, and occupational asthmagens—that collectively account for approximately 30% of the global disease burden (2).

For decades, asthma pathogenesis research has centered on a T cell-centric immunological model, emphasizing the role of Th2 cells and their signature cytokines—IL-4, IL-5, and IL-13 (3)—in orchestrating eosinophilic airway inflammation. This paradigm successfully explained many features of allergic asthma and facilitated the development of targeted biologics against IL-5 and IL-4/IL-13 pathways (4). However, this traditional framework offers incomplete mechanistic explanations for several important features of asthma, prompting broader re-evaluation of disease mechanisms.

First, the T cell-centric model cannot adequately explain the chronicity and recurrent nature of asthma—why does airway inflammation persist even during periods of apparent clinical remission and in the absence of continuous allergen exposure? (5) Second, in severe asthma cohorts—including the SARP (Severe Asthma Research Program) and U-BIOPRED registries, where T2-low phenotypes are systematically overrepresented relative to general asthma populations—an estimated 30%–50% of patients are classified as T2-low based on the absence of elevated blood eosinophils and FeNO (6, 7)—a heterogeneous phenotype encompassing neutrophilic, paucigranulocytic, and mixed granulocytic inflammation, as well as obesity-related and late-onset clinical subtypes (7). T2-low asthma is generally associated with corticosteroid insensitivity and a lack of approved biologic therapies, although tezepelumab has demonstrated efficacy across T2 biomarker strata (6). Prevalence estimates vary substantially with the biomarker thresholds applied (blood eosinophils <150 vs. <300 cells/µL; FeNO <20 vs. <25 ppb), the population studied, and whether corticosteroid-mediated biomarker suppression has been accounted for; moreover, T2-low status is temporally unstable, with biomarker reclassification occurring in up to 45% of patients on longitudinal follow-up (8).Third, the traditional model struggles to explain why early-life exposures—including viral infections, air pollution, and tobacco smoke—exert lasting effects on asthma risk that persist for decades (9).

### 1.2 Airway epithelium, epigenetic regulation, and epithelial memory as a unifying framework

These limitations have prompted a shift from viewing the airway epithelium as a passive barrier to recognizing it as a central organizer of asthma pathogenesis. The epithelium is strategically positioned at the interface between host and environment, where it senses inhaled allergens, pollutants, and microbes, coordinates early immune responses, and helps determine whether airway homeostasis is restored or replaced by persistent dysfunction (10, 11).This perspective is supported by the observation that many asthma susceptibility pathways converge on epithelial barrier integrity, injury responses, and epithelial-immune crosstalk (12).

A second key insight is that epithelial dysfunction in asthma is not simply transient. Environmental and inflammatory exposures can induce durable molecular changes through epigenetic mechanisms, including DNA methylation, histone modifications, and non-coding RNAs (13). These mechanisms provide a plausible link between external exposures and persistent alterations in epithelial behavior, allowing short-lived insults to generate long-lasting effects on barrier function, inflammatory responsiveness, and tissue repair (14).

**Figure 1:**
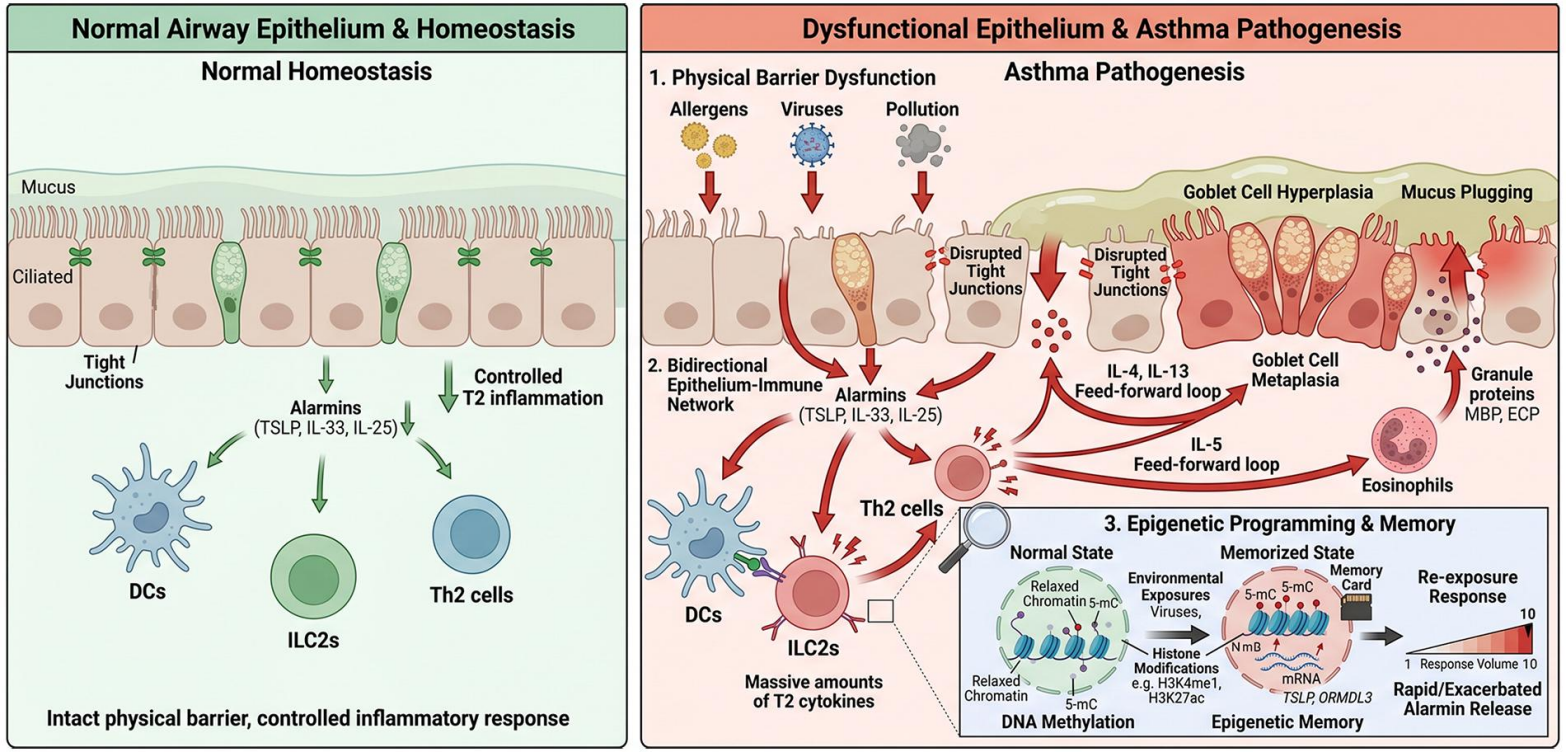
The bidirectional interplay between airway epithelial dysfunction, immune networking, and epigenetic memory in asthma pathogenesis. In healthy airways (left panel), an intact epithelial barrier maintains immune homeostasis. Upon exposure to environmental triggers (right panel), disrupted junctional integrity leads to the release of alarmins (TSLP, IL-33, IL-25), which activate innate (ILC2s, dendritic cells) and adaptive (Th2) immune cells. A self-amplifying, bidirectional loop is established as Th2-derived cytokines (IL-4, IL-13) reciprocally downregulate tight junction proteins to further exacerbate barrier leakage and alarmin release. Crucially, repeated inflammatory insults induce durable epigenetic reprogramming—characterized by DNA methylation changes, histone modifications, and miRNA dysregulation—within basal progenitor cells. This “epigenetic memory” sustains barrier dysfunction and hyperresponsiveness even after acute inflammation resolves. Current and emerging therapies, including upstream anti-alarmin biologics (tezepelumab, itepekimab), downstream cytokine antagonists (dupilumab), and epigenetic modifiers (HDAC inhibitors), target distinct nodes within this pathological circuit.

This framework incorporates the concept of epithelial memory, in which prior exposures leave lasting molecular imprints that shape future epithelial responses (5). In asthma, such memory may help explain chronicity, recurrence after apparent remission, and the lasting influence of early-life exposures. Accordingly, this review considers asthma not only as a disorder of dysregulated immunity, but also as a disease of maladaptively reprogrammed barrier tissue. The following sections examine how epithelial dysfunction emerges, how it is stabilized by epigenetic programming, and how these processes may be therapeutically targeted. The following sections examine how epithelial dysfunction emerges, how it is stabilized by epigenetic programming, and how these processes may be therapeutically targeted (Figure 1).

## 2 Part II: Airway epithelial dysfunction as the initiating platform

### 2.1 Barrier architecture and junctional defects

The airway epithelium constitutes a pseudostratified structure comprising multiple specialized cell types that collectively form the first line of defense against inhaled environmental insults (15). Ciliated cells, the most abundant epithelial cell type, propel mucus and trapped particles toward the pharynx through coordinated ciliary beating, while goblet cells and submucosal glands produce mucus that traps pathogens for subsequent expulsion (16). Basal cells serve as progenitor cells, differentiating into various epithelial cell types to maintain homeostasis following injury (17). The epithelium performs three critical barrier functions: physical (maintained by intercellular junctional complexes), functional (mucociliary clearance), and immunological (detection of environmental threats through pattern recognition receptors and orchestration of immune responses).

Tight junction dysfunction represents a hallmark of asthmatic airway epithelium and plays a central role in disease pathogenesis (18). Tight junctions form a barrier on the apical side of neighboring epithelial cells, and changes in their integrity enable the paracellular influx of allergens, toxins, and microbes to the submucosal tissue (19). Bronchial epithelial cells from asthmatic patients demonstrate significantly reduced tight junction integrity in air-liquid interface cultures compared with cells from healthy subjects, establishing that barrier dysfunction is an intrinsic property of asthmatic epithelium rather than merely a consequence of ongoing inflammation (20). Type 2 cytokines IL-4 and IL-13 induce physical separation of tight junctions between adjacent cells, as demonstrated by disrupted immunofluorescence staining of the tight junction molecules occludin and zonula occludens-1 (ZO-1) (21).

Claudin-18, the only known lung-specific tight junction protein, has emerged as a critical mediator of epithelial barrier dysfunction in asthma. Epithelial brushings from patients with asthma have significantly lower *CLDN18* mRNA levels than those from healthy controls, with the lowest levels observed in T2-high patients (22). IL-13 decreases claudin-18 expression in primary human cells and in mice, and *Cldn18*-null mice exhibit higher serum IgE levels and increased airway responsiveness following allergen sensitization. Differential expression of tight junction molecules across asthma phenotypes has been demonstrated: ZO-1 and claudin-18 are downregulated in all phenotypes (eosinophilic, mixed, and neutrophilic), while increased claudin-4 expression is characteristic of neutrophilic airway inflammation (23).

E-cadherin, an adherens junction protein critical for epithelial integrity, plays a pivotal role in maintaining airway homeostasis. Studies using a lung epithelium-specific E-cadherin knockout mouse model demonstrated that loss of this junction protein leads to progressive epithelial damage including airway epithelial denudation, decreased ZO-1 expression, and loss of ciliated cells (24). It should be noted that this murine model also developed enlarged alveolar spaces—a feature more characteristic of COPD than asthma—indicating that the consequences of complete E-cadherin ablation extend beyond an asthma-specific phenotype (25). Nevertheless, the spontaneous goblet cell metaplasia, eosinophil infiltration, and elevated CCL17 levels observed support the conclusion that adherens junction disruption is sufficient to initiate a type 2-dominant inflammatory cascade (26).

Importantly, E-cadherin downregulation activates EGFR-dependent signaling that promotes type 2 immune cell recruitment. In human bronchial epithelial cells, loss of E-cadherin-mediated cell contact results in enhanced phosphorylation of EGFR and downstream targets including MEK/ERK-1/2 and p38 MAPK, ultimately upregulating expression of the Th2 cell-attracting chemokine TARC and the alarmin TSLP (27). This mechanism, characterized directly in asthmatic airway epithelial cells, establishes a causal link between adherens junction disruption and the initiation of type 2 inflammation. A complementary line of evidence comes from a study of chronic obstructive airway disease models, in which selective inactivation of E-cadherin in epithelial progenitor club cells—but not in terminally differentiated ciliated cells—led to increased epithelial proliferation, goblet cell metaplasia, and immune cell infiltration (26). While this study was conducted in the context of obstructive lung disease broadly rather than asthma specifically, the phenotypes induced by progenitor-cell E-cadherin loss—particularly goblet cell hyperplasia and inflammatory infiltration in the absence of allergen—are mechanistically relevant to asthma and suggest that the pathogenic role of E-cadherin disruption in epithelial progenitor populations may extend across obstructive airway diseases, though direct evidence in asthmatic club cells independent of cigarette smoke exposure is lacking (28).

### 2.2 Epithelial inflammatory circuits and mucus pathology

Type 2 innate lymphoid cells (ILC2s) have emerged as critical mediators of bronchial epithelial barrier disruption through their production of IL-13. ILC2s significantly impair the epithelial barrier, as demonstrated by reduced transepithelial electrical resistance and increased permeability in air-liquid interface cultures of human bronchial epithelial cells (29).This barrier impairment is paralleled by decreased mRNA and disrupted protein expression of tight junction proteins, which is restored by IL-13 neutralization, establishing a direct causal relationship between ILC2-derived IL-13 and epithelial barrier dysfunction.

*In vivo* studies confirm this mechanism through elegant genetic approaches. Intranasal administration of IL-33 to wild-type and *Rag2* mice (lacking T and B cells) triggers tight junction disruption, whereas *Rag2*/*Il2rg* and *Rora* mice (lacking ILC2s) do not show barrier leakiness despite IL-33 exposure (29). Direct nasal administration of IL-13 is sufficient to induce tight junction barrier deficiency in the bronchial epithelium, demonstrating that IL-13 alone can recapitulate the barrier-disrupting effects of ILC2 activation. ILC2s respond rapidly to epithelium-derived alarmins (IL-33, IL-25, TSLP) and produce type 2 effector cytokines that characterize the pathology of allergic asthma (30, 31); their activation may also confer steroid resistance.

Mucus plugging is a central pathophysiological feature of asthma that has been recognized by pathologists for over 100 years, yet is often underappreciated clinically. The two major gel-forming mucins in the airways are MUC5AC and MUC5B, and in asthma, there is a pathological shift toward MUC5AC dominance that has profound functional consequences (32). IL-13 stimulation induces the formation of heterogeneous mucus gels and dramatically impairs mucociliary transport through a mechanism that does not involve ciliary dysfunction (33). Rather, the impairment results from tethering of MUC5AC-containing mucus gel domains to mucus-producing cells in the epithelium (34); replacement of tethered mucus with untethered mucus restores mucociliary transport, indicating that MUC5AC tethering causes mucostasis and likely represents a major cause of mucus plugging in asthma (35).

Genetic studies have demonstrated that *MUC5AC* gene is required for allergic airway hyperreactivity, establishing a causal rather than merely correlative relationship. In mice bred on two well-characterized strain backgrounds and exposed to two separate allergic stimuli, genetic removal of *Muc5ac* abolishes airway hyperreactivity. Residual methacholine responses are identical to unchallenged controls, and although inflammation remains intact, heterogeneous mucous occlusion decreases by 74% (36). Thus, whereas inflammatory effects on airway smooth muscle alone are insufficient for airway hyperreactivity, *Muc5ac*-mediated plugging is an essential mechanism.

The alarmin cytokines TSLP, IL-33, and IL-25 are epithelial cell-derived mediators that contribute fundamentally to asthma pathobiology by bridging environmental exposures to downstream inflammatory cascades (37). Released from airway epithelial cells exposed to allergens, viruses, pollutants, and other environmental triggers, the alarmins drive airway inflammation through the release of predominantly type 2 cytokines from multiple effector cells including ILC2s, mast cells, basophils, dendritic cells, Th2 cells, and select regulatory T cell subsets that express cognate alarmin receptors such as ST2 (IL-33 receptor), TSLPR, and IL-17RB. The upstream positioning of the alarmins in the inflammatory cascade makes them attractive pharmacological targets (38). TSLP conditions dendritic cells to initiate type 2 responses and is particularly potent in activating ILC2s (39). IL-33 directly activates mast cells and ILC2s and may influence asthma susceptibility through its role in establishing the immune environment in perinatal lungs (40). Despite considerable overlap in cellular functions, these alarmins appear to have distinct roles in asthma immunopathology: a TSLP/ILC axis may mediate steroid resistance (41), while memory Th2 cell subsets characterized by high receptor expression for these cytokines support a role in allergic exacerbations (42).

Critically, the relationship between the airway epithelium and the adaptive immune compartment is bidirectional rather than unidirectional. Epithelial alarmin release primes T cell polarization, while T cell-derived IL-4 and IL-13 in turn perpetuate the barrier disruption that sustains alarmin secretion (43). This epithelial–Th2 feedforward loop functions as a self-amplifying circuit rather than a linear cascade, providing the mechanistic rationale for targeting the epithelial level to interrupt disease chronicity (44) (Figure 1).

### 2.3 Genetic susceptibility and single-cell evidence for epithelial heterogeneity

Many asthma susceptibility loci identified by genome-wide association studies are preferentially expressed in the airway epithelium, reinforcing the concept that disease initiation is rooted, at least in part, at the epithelial surface. The 17q12-21 locus, including *ORMDL3* and *GSDMB* (45), remains one of the most consistently replicated genetic associations, particularly in childhood asthma, while epithelial-related genes such as *IL33*, *TSLP*, *IL1RL1*, *IL7R*, and *CDHR3* further support a model in which barrier regulation, antiviral defense, and epithelial-immune signaling are central to disease susceptibility. Gene-environment interactions strengthen this view, as the effects of several risk variants are modified by early-life viral exposures, tobacco smoke, and other environmental factors (46)—suggesting that genetic predisposition and environmental insult converge on the epithelium rather than operating through independent pathways.

Single-cell and spatial transcriptomic studies have added an important cellular dimension to these genetic observations by demonstrating that the asthmatic epithelium is not merely inflamed, but actively reconfigured into distinct disease-associated cell states (47). These studies have identified aberrant epithelial populations—including mucous ciliated cells and MUC5AC-enriched secretory niches—that help explain why mucus dysfunction in asthma cannot be reduced to simple goblet cell expansion alone (48). They also reveal depletion of protective epithelial programs, altered repair trajectories, and remodeling of cell-cell communication toward a type 2-dominant interactome following allergen exposure (49). Multicohort transcriptomic analyses have further identified reproducible epithelial expression clusters associated with asthma severity, lung function, bronchodilator reversibility, and type 2 inflammation, suggesting that epithelial endotypes carry meaningful clinical correlates (50). Together, these data establish the airway epithelium as a biologically heterogeneous, disease-defining compartment rather than a passive cellular lining (51).

Critically, these epithelial reconfigurations persist across disease states and in the absence of continuous allergen exposure—a pattern inconsistent with transient inflammatory activation alone (14). This persistence implies the existence of stable molecular imprints that lock the epithelium into a disease-associated state, directing attention toward epigenetic mechanisms as the molecular basis of airway epithelial chronicity in asthma (52).

## 3 Part III: Epigenetic programming of the asthmatic epithelium

### 3.1 DNA methylation

DNA methylation involves the addition of a methyl group to specific cytosines, producing 5-methylcytosine, and this process occurs most frequently at cytosine-phosphate-guanine (CpG) sites (53). Hypermethylation of gene promoter regions typically leads to transcriptional silencing, while hypomethylation is generally associated with increased gene expression. Because epigenetic marks can be modified by both environmental exposures and genetic variation, they are increasingly recognized as relevant to asthma pathogenesis and may serve as a key link between environmental exposures and disease susceptibility (54).

It should be noted that several EWAS discussed below were conducted in peripheral blood or cord blood rather than airway epithelial cells; where relevant, we discuss the biological plausibility of cross-tissue epigenetic convergence. Epigenome-wide association studies have identified substantial DNA methylation differences in airway epithelial cells from asthmatic patients compared with healthy controls (55). In a landmark study of nasal epithelium from children with persistent atopic asthma, 186 genes showed significant methylation changes, with a median methylation difference of 9.5% (56). Differentially methylated genes included those with established roles in asthma and atopy, as well as genes related to extracellular matrix, immunity, cell adhesion, and epigenetic regulation. Importantly, hypomethylated and hypermethylated genes were associated with increased and decreased gene expression, respectively, confirming the functional relevance of these epigenetic marks.

A whole-genome bisulfite sequencing study of peripheral blood from asthmatic children identified 158 differentially methylated regions, revealing global hypomethylation preferentially affecting enhancer regions and associated with aberrant expression of *IL4*, *IL5RA*, and *EPX*—genes directly relevant to eosinophilic airway inflammation (57). Notably, 56 of these regions were already perturbed at birth and linked to prenatal tobacco smoke or phthalate exposure, suggesting that epigenetic programming relevant to asthma susceptibility begins *in utero* (58).

Epigenome-wide meta-analysis conducted primarily in neonatal cord blood and pediatric peripheral blood identified 9 CpGs and 35 differentially methylated regions in newborns prospectively associated with asthma development, and 179 CpGs and 36 regions in cross-sectional analysis of older children. Prospective cord blood associations are particularly informative as they capture epigenetic determinants established before disease onset (59).

Importantly, a substantial proportion of CpGs identified in adult peripheral blood replicated in nasal respiratory epithelial cells and eosinophils (60), supporting cross-tissue relevance. Several implicated genes are targets for approved or experimental drugs, including *IL5RA* and *KCNH2* (61).

Aberrant methylation of barrier-related genes, including filaggrin (*FLG*) and claudins (*CLDNs*), disrupts tight junctions and enhances allergen penetration (62). Expression quantitative trait methylation analysis has revealed that most methylation probes affecting gene expression are located distantly from their target genes (average distance approximately 378 kb) and are more likely to be located in enhancer regions, suggesting that distant epigenetic regulation of gene expression in airway epithelium plays a significant role in atopic asthma (63).

### 3.2 Histone modifications

Histone modifications represent another critical layer of epigenetic regulation in asthma (64). Histones can undergo various post-translational modifications, including acetylation, methylation, phosphorylation, and ubiquitination, which alter chromatin accessibility and gene transcription (65). Histone acetylation, catalyzed by histone acetyltransferases (HATs) and removed by histone deacetylases (HDACs), is particularly relevant to asthma pathogenesis.

The airway epithelium from asthmatic subjects displays increased acetylation of histone 3 lysine 18 (H3K18ac) and trimethylation of histone 3 lysine 9 (H3K9me3) compared with healthy subjects (52). Increased association of H3K18ac around the transcription start sites of *ΔNP63*, *EGFR*, and *STAT6*—genes integral to epithelial differentiation, proliferation, and inflammation—has been observed in airway epithelial cells from asthmatics, suggesting a complex interaction between histone modifications and gene regulation in asthma.

A critical discovery linking histone modifications to epithelial barrier dysfunction is the finding that HDAC expression is significantly elevated in bronchial epithelial cells from asthmatic patients. *HDAC1*, *HDAC9*, *SIRT6*, *SIRT7* expression is higher in asthmatic epithelium, and IL-4 and IL-13 further increase HDAC and SIRT. Importantly, HDAC inhibition improves barrier integrity through increased synthesis of tight junction molecules in asthmatic epithelium to levels seen in healthy controls. This finding establishes a direct mechanistic link between epigenetic regulation and epithelial barrier dysfunction, demonstrating that barrier leakiness in asthmatic patients is induced by T2 cells, IL-4, IL-13, and HDAC activity (18).

The mechanisms by which HDACs regulate tight junction expression involve chromatin remodeling at the promoters of junctional genes. Histone acetylation generally promotes gene transcription by relaxing chromatin structure, while deacetylation by HDACs leads to chromatin condensation and transcriptional repression (66). The elevated HDAC activity in asthmatic epithelium thus creates a chromatin environment unfavorable for tight junction gene expression, perpetuating barrier dysfunction even in the absence of ongoing inflammatory stimulation (18).

### 3.3 Non-coding RNAs

Non-coding RNAs, including microRNAs (miRNAs) and long non-coding RNAs (lncRNAs), represent a third major class of epigenetic regulators in asthma (67). MicroRNAs are small (approximately 22 nucleotides) non-coding RNAs that regulate gene expression post-transcriptionally by binding to complementary sequences in target mRNAs, leading to translational repression or mRNA degradation (68).

Dramatic alterations of airway epithelial cell miRNA levels are a common feature of asthma. In a comprehensive study of bronchial epithelial brushings, 217 miRNAs were differentially expressed in steroid-naive subjects with asthma compared with controls, and 200 miRNAs were differentially expressed in steroid-using subjects with asthma (69). Notably, treatment with inhaled corticosteroids had only modest effects on miRNA expression, inducing statistically significant changes for only nine miRNAs, suggesting that miRNA dysregulation in asthma is relatively resistant to conventional therapy.

Analysis of bronchial biopsies identified 78 differentially expressed miRNAs in asthma patients compared with controls, of which 60 remained differentially expressed after controlling for smoking and inhaled corticosteroid treatment (70). Several asthma-associated miRNAs were identified, including miR-125b-5p and miR-223-3p, based on significant associations with multiple clinical and inflammatory asthma features (71). The most enriched biological pathways affected by these miRNAs were inflammatory response and cilium assembly/organization. A hub of six dysregulated microRNAs was found to account for approximately 90% of all microRNA targeting in severe asthma, and transfection of this hub in bronchial epithelial cells from healthy donors mimicked asthma characteristics (72).

Specific miRNAs regulate epithelial barrier function. MiR-363-3p, induced by IL-17A and TNF*α* stimulation mimicking Th17 inflammation, contributes to epithelial damage by targeting and suppressing gene expression of several key barrier components, including *CLDN8*, *PCDH1*, and *PTEN* (73). Similarly, exosomal miR-129-2-3p promotes airway epithelial barrier disruption in PM2.5-aggravated asthma by targeting the TIAM1/RAC1/PAK1 signaling pathway (74).

Long non-coding RNAs have also emerged as important regulators in asthma. Bioinformatics analysis has identified a lncRNA-miRNA-mRNA regulatory network in severe asthmatic bronchial epithelial cells, with the top competing endogenous RNAs including *FGD5-AS1*, *MALAT1*, *XIST*, *HCG18*, *SNHG16* (75). These lncRNAs regulate key pathways including MAPK, Rap1, Ras, PI3K-Akt, and calcium signaling pathways. The lncRNA plasmacytoma variant translocation 1 (PVT1) is decreased in patients with corticosteroid-sensitive nonsevere asthma and increased in patients with corticosteroid-insensitive severe asthma, suggesting that targeting this lncRNA might be effective in reducing airway remodeling (76).

Collectively, the scale of ncRNA dysregulation in asthmatic airway epithelium—encompassing hundreds of differentially expressed species organized into functionally coherent hubs—and its broad resistance to normalization by inhaled corticosteroids suggest that ncRNA-mediated regulation operates as a relatively autonomous layer of epithelial pathological programming (69). This has direct therapeutic implications: even when upstream cytokine signaling is suppressed, residual ncRNA imbalances may sustain barrier dysfunction, altered differentiation, and mucus hypersecretion, underscoring the potential of ncRNA-targeted interventions as a complement to conventional anti-inflammatory therapy (68).

### 3.4 Environmental exposures and developmental programming

Environmental factors play a crucial role in establishing epigenetic marks that influence asthma susceptibility. The sharp increase in asthma prevalence over the past two to three decades and the large variations among populations of similar racial/ethnic background but different environmental exposures favor a strong contribution of environmental factors mediated through epigenetic mechanisms (77).

Tobacco smoke exposure represents one of the most well-characterized environmental influences on the asthma epigenome. Prenatal tobacco smoke exposure induces consistent DNA methylation changes, particularly at the aryl hydrocarbon receptor repressor gene (AHRR), that are associated with increased asthma risk (78). Air pollution, including particulate matter (PM2.5), diesel exhaust particles, and polycyclic aromatic hydrocarbons, also induces epigenetic modifications that contribute to airway epithelial dysfunction and asthma pathogenesis (79).

A critical insight from human exposure studies is that the bronchial epithelium does not respond to environmental insults in isolation—rather, prior exposures can fundamentally alter how the epithelium responds epigenetically to subsequent challenges. Clifford and colleagues examined this phenomenon in a randomized crossover-controlled human inhalation study, in which participants were exposed to allergen alone, diesel exhaust alone, or both simultaneously. When these exposures occurred within a single occasion, only 7 CpG sites showed significant methylation changes at 48 h (80). However, when the same participants underwent sequential exposures separated by approximately four weeks—first one insult, then the other—more than 500 CpG sites were differentially methylated, representing a more than 70-fold amplification in epigenetic response magnitude. Moreover, the specific sites affected depended on the order in which exposures were experienced, demonstrating that the epigenetic consequences of environmental co-exposure are not additive but contextually determined by prior exposures.

These findings suggest that the bronchial epithelium maintains an exposure history that actively shapes its future epigenetic responsiveness—a form of environmental priming that provides a mechanistic basis for the concept of epithelial epigenetic memory in asthma.

Prenatal exposure to particulate matter has been associated with DNA methylation changes in newborns at CpGs mapped to genes previously associated with lung function and asthma, including *FAM13A* and *NOTCH4* (81). Maternal prenatal immune status shapes asthma development in offspring by altering the epigenome and trained innate immunity at birth. A module of differentially methylated CpG sites enriched for microbe-responsive elements was associated with childhood asthma, and *in vitro* responsiveness to microbial products was impaired in cord blood mononuclear cells from neonates who later developed asthma (82).

Epigenome-wide analysis in neonatal cord blood mononuclear cells has linked *SMAD3* methylation at birth to asthma risk in children of asthmatic mothers. Methylation at this locus was selectively increased in asthmatic children born to asthmatic mothers and replicated across multiple independent cohorts. Moreover, *SMAD3* methylation levels were strongly correlated with neonatal production of IL-1β, an innate inflammatory mediator central to airway mucosal responses (83). Although this study was conducted in cord blood rather than airway epithelial cells, SMAD3 is a key transducer of TGF-β signaling in airway epithelial biology; its epigenetic silencing in the neonatal period may therefore reflect a systemic predisposition toward impaired epithelial repair and exaggerated inflammatory responsiveness that subsequently manifests at the airway mucosal surface (84). Grandmaternal allergen sensitization during pregnancy can reprogram epigenetic and airway responses to allergen in second-generation offspring, demonstrating transgenerational epigenetic inheritance of asthma susceptibility (85).

### 3.5 Divergent epigenetic and inflammatory landscapes in T2-high versus T2-low asthma

T2-low asthma encompasses a heterogeneous group of endotypes characterized by absent or low type 2 biomarker signals, frequent neutrophilic or paucigranulocytic airway inflammation, and poor responsiveness to inhaled corticosteroids and current biologics targeting IL-4/IL-13 or IL-5 pathways. Emerging data suggest that airway epithelial dysfunction is also central in T2-low disease, but with distinct features compared with T2-high asthma. Transcriptomic profiling of nasal epithelial cells from pediatric asthma cohorts has identified molecular endotypes that parallel clinical and inflammatory asthma subtypes, with cluster analysis of T2 and T17 signature genes revealing three distinct profiles—T2-high, T17-high, and T2-low/T17-low—that are reproducible across cohorts of racially and ethnically diverse youths (86). T2-high asthma is characterized by enhanced T2 immune responses reflected in elevated serum T2 cytokines (IL-4, IL-5, IL-13), total IgE, and airway eosinophils, while T2-low patterns show distinct transcriptomic signatures (87). Using a transcriptomic method applied to induced sputum and peripheral blood, Peters and colleagues differentiated T2-low asthma from healthy controls by lower expression of a cytotoxic CD8⁺ T-cell gene network negatively correlated with body mass index and circulating IL-6 concentrations (88), suggesting that obesity-related systemic inflammation may suppress cytotoxic T-cell surveillance in the T2-low airway.

#### 3.5.1 Epigenetic distinctions

At the epigenetic level, T2-high and T2-low asthma harbor fundamentally distinct chromatin programs rooted in their divergent inflammatory microenvironments. In T2-high disease, IL-4/IL-13-driven upregulation of HDAC1, HDAC9, SIRT6, and SIRT7 creates a chromatin environment that represses tight junction gene transcription, imprinting a pro-leaky barrier state that persists even after cytokine withdrawal (18). Concurrently, IL-13-induced DNA methylation changes are selectively enriched near asthma susceptibility genes, establishing durable molecular scars that sustain goblet cell metaplasia, MUC5AC overexpression, and barrier hyper-permeability beyond the period of active cytokine stimulation (89). In T2-low disease, a mechanistically divergent epigenetic axis operates: oxidative stress from cigarette smoke, ozone, and occupational exposures impairs HDAC2 activity, disrupting glucocorticoid receptor deacetylation and thereby directly reducing corticosteroid responsiveness (90)—an axis largely absent in T2-high disease. Methylation signatures in airway cells from adult children of asthmatic mothers further reflect T2-low-specific epigenetic vulnerability, with a maternal asthma-associated methylation module enriched for pathways reflecting impaired T cell signaling and responses to viral and bacterial pathogens correlating specifically with low total serum IgE, FeNO, and eosinophilia (91). Non-coding RNA profiles also diverge: miR-363-3p, induced by IL-17A and TNF-α signaling characteristic of T2-low neutrophilic inflammation, targets and suppresses *CLDN8*, *PCDH1*, and *PTEN*, producing a pattern of barrier disruption mechanistically distinct from IL-13-driven ncRNA dysregulation in T2-high disease (73); the lncRNA PVT1, elevated specifically in corticosteroid-insensitive severe asthma, further exemplifies a T2-low-enriched epigenetic feature with direct clinical relevance (76).

#### 3.5.2 Upstream inflammatory architecture

These endotype-specific epigenetic programs do not arise in isolation—they are generated and sustained by fundamentally divergent upstream inflammatory circuits. T2-high asthma is defined by ILC2- and Th2-orchestrated eosinophilic inflammation driven by IL-4, IL-5, IL-13, and elevated IgE, FeNO, and periostin reflecting ongoing IL-13-mediated epithelial activation (92). T2-low disease, by contrast, is sustained by innate immune pathways largely independent of adaptive T2 immunity, dominated by IL-17A, IL-8, TNF-α, and IL-1β (6). IL-17A promotes neutrophil recruitment via CXCL1/CXCL5 induction and synergizes with TNF-α to amplify NF-κB-driven epithelial injury, while NLRP3 inflammasome activation—triggered by ATP, uric acid, and particulate pollutants—drives caspase-1-dependent IL-1β/IL-18 secretion that sustains corticosteroid-insensitive neutrophilic inflammation (93). Obesity-associated T2-low asthma introduces further complexity through adipokine dysregulation: elevated leptin and reduced adiponectin amplify NF-*κ*B-driven epithelial alarmin secretion while suppressing regulatory T cell function, and a mechanically restrictive chest wall compounds airway hyperresponsiveness independently of eosinophilic inflammation (7). In paucigranulocytic T2-low asthma, smooth muscle hyperreactivity and airway remodeling appear to persist in the near-absence of granulocytic infiltration, suggesting that epigenetically fixed structural abnormalities can sustain airway dysfunction autonomously—a dissociation between inflammation and remodeling with important implications for therapeutic design.

#### 3.5.3 Convergence at the alarmin level and therapeutic implications

Despite this mechanistic divergence, a partial point of convergence exists at the level of epithelial alarmin release. TSLP is induced not only by IL-4 and IL-13 but also by IL-17A, NF-*κ*B activation, and oxidative stress—stimuli predominant in T2-low disease—placing it upstream of the T2-high/T2-low divergence point (94). This cross-endotype TSLP expression provides the mechanistic rationale for the pan-endotype efficacy of tezepelumab observed in the NAVIGATOR trial (94), and simultaneously illustrates why downstream T2 cytokine blockade (dupilumab, mepolizumab) offers limited benefit in T2-low disease: these agents act downstream of the divergence point and cannot intercept the NF-κB/NLRP3-driven inflammatory circuits that sustain the T2-low airway. The distinct innate immune architecture of T2-low asthma thus both explains the current therapeutic gap and identifies specific intervention nodes—the IL-17A/NLRP3 axis, HDAC2 restoration, and reprogramming of innate epigenetic memory—that warrant dedicated clinical investigation, as discussed in Part V.

The key mechanistic and therapeutic distinctions between T2-high and T2-low asthma endotypes are summarized in Table 1.

**Table 1:** Mechanistic and therapeutic distinctions between T2-high and T2-low asthma endotypes.

Feature	T2-high asthma	T2-low asthma
Dominant inflammation	Eosinophilic	Neutrophilic/paucigranulocytic
Key cytokines	IL-4, IL-5, IL-13, IL-33, TSLP	IL-17A, IL-8, TNF-α, IL-1β
Innate immune amplifier	ILC2, mast cell	NLRP3 inflammasome, NF-*κ*B
Primary trigger	Allergens, viral infection	Smoke, pollution, obesity, non-atopic stimuli
Epigenetic hallmark	HDAC1/9↑, IL-13 methylation scars, basal cell allergic memory	HDAC2↓, NF-κB/innate immune gene hypomethylation, PVT1↑
Corticosteroid response	Generally responsive	Reduced/resistant
Effective biologics	Dupilumab, mepolizumab, benralizumab, tezepelumab	Tezepelumab (partial); no approved T2-low-specific biologic
Emerging therapeutic targets	HDAC1/9 inhibition, miR-21/miR-145 antagomirs	HDAC2 restoration, NLRP3 inhibition, anti-IL-17A, miR-223-3p modulation

## 4 Part IV: Epithelial memory and trained immunity

### 4.1 Conceptual basis of epithelial memory

A fundamental question in asthma pathogenesis is why airway epithelial dysfunction persists even after the resolution of acute inflammation or removal of environmental triggers (95). The concept of “epithelial memory” provides a compelling explanation (52): cells can maintain altered epigenetic states long after the initial stimulus has been removed, resulting in lasting changes in gene expression and cellular function.

Emerging data suggest that the airway epithelium in asthma remembers prior encounters with environmental exposures, resulting in potentially long-lasting changes in structure and metabolism that render asthmatic individuals susceptible to subsequent exposures (96). The history of allergy- and pathogen-derived insults can leave some kind of memory or “imprint” in airway epithelial cells, placing the airway epithelium at the center of processes that lead to formation, progression, and acute exacerbation of asthma. Unlike adaptive immune memory mediated by T and B cells, epithelial memory operates through epigenetic and metabolic reprogramming that does not require antigen-specific receptors.

The key distinctions between adaptive immune memory and epithelial/innate immune memory are summarized in Table 2.

**Table 2:** Key distinctions between adaptive immune memory and epithelial/innate immune memory.

Feature	Adaptive immune memory	Epithelial/innate immune memory
Cell types	T cells, B cells	Epithelial cells, macrophages, ILC2s
Recognition	Antigen-specific receptors	Pattern recognition receptors, epigenetic priming
Molecular basis	Clonal expansion, receptor recombination	DNA methylation, histone modifications, metabolic reprogramming
Specificity	High (antigen-specific)	Broad (cross-reactive to multiple stimuli)
Reversibility	Largely irreversible	Potentially reversible through epigenetic modulation

This concept provides an entirely new perspective for understanding the chronicity and recurrence of asthma, explaining why asthmatic airways maintain a hyperresponsive state even during periods of apparent clinical remission, and why early-life exposures can have lasting effects on disease susceptibility decades later.

### 4.2 IL-13-Induced persistent reprogramming

The type 2 cytokine IL-13 has emerged as a key mediator of epigenetic memory in asthmatic airways. A critical mechanistic insight is that IL-13 does not merely alter epithelial gene expression acutely—it can imprint durable DNA methylation changes that outlast the cytokine exposure itself. Genome-wide methylation profiling of primary airway epithelial cell cultures demonstrates that IL-13-induced methylation shifts are selectively enriched near asthma susceptibility genes, suggesting that cytokine-driven epigenetic remodeling is not a non-specific response to cellular stress but a targeted reprogramming of disease-relevant loci. The biological relevance of these *in vitro* marks is reinforced by their partial replication in freshly isolated airway epithelial cells from asthmatic donors—an alignment between cultured and *in vivo* epigenetic states that supports the existence of IL-13-imprinted chromatin scars under conditions of chronic type 2 inflammation (89).

Importantly, the IL-13-associated methylation landscape is not monolithic. Network-based coexpression analysis resolves it into at least two functionally distinct modules: one tracking clinical disease severity and lung function trajectories, and another co-varying with eosinophilic burden (89). This modular architecture implies that IL-13 does not produce a single uniform epigenetic scar but rather calibrates distinct chromatin programs according to the inflammatory context—an observation with direct implications for biomarker development, as it raises the possibility of epigenetic signatures that independently predict airway obstruction progression vs. eosinophilic relapse risk.

Beyond reshaping DNA methylation, IL-13 also drives broad transcriptional reprogramming in airway epithelial cells that is tightly coupled to chromatin remodeling. IL-13 stimulation activates transcription factors such as STAT6 and SPDEF and recruits co-activators including CREB-binding protein (CBP), which catalyze histone H3 acetylation at promoters and enhancers of IL-13-responsive genes. In airway fibroblasts derived from allergic inflammation models, markedly increased global acetylation of histone 3 has been observed (97); while this finding derives from a non-epithelial cell type, it suggests a broader chromatin-level permissiveness within the inflamed airway microenvironment that may extend to epithelial compartments, though direct epithelial confirmation is warranted. IL-13 stimulation also reveals the remarkable cellular plasticity of the airway epithelium: both *in vivo* murine models and human mucociliary airway epithelial cultures have shown that IL-13 induces cellular remodeling through proliferation-independent transdifferentiation, resulting in generation of hypersecretory MUC5AC-expressing mucus cells and loss of ciliated cells (33)—plasticity that is potentially adaptive in acute settings but becomes maladaptive in the context of chronic type 2 inflammation. Together, these findings support a model in which episodic IL-13 exposure not only acutely alters epithelial structure and function, but also imprints long-lasting epigenetic signatures that contribute to the chronicity and relapse-prone behavior of asthmatic airways.

### 4.3 Basal progenitors as repositories of allergic memory

A groundbreaking discovery in understanding epithelial memory came from single-cell RNA sequencing studies of human respiratory epithelium in chronic rhinosinusitis, a type 2 inflammatory disease closely related to asthma (98). These studies revealed that basal cells, which serve as progenitor cells for the airway epithelium, can retain intrinsic memory of IL-4/IL-13 exposure. Single-cell profiling revealed marked differences between the epithelial compartments of non-polyp and polyp cellular ecosystems, identifying a global reduction in cellular diversity characterized by basal cell hyperplasia, concomitant decreases in glandular cells, and phenotypic shifts in secretory cell antimicrobial expression. An aberrant basal progenitor differentiation trajectory was detected in polyps, with cell-intrinsic, epigenetic, and extrinsic factors proposed to lock polyp basal cells into an uncommitted state. Critically, *ex vivo* cultured basal cells from polyp tissue were shown to retain intrinsic memory of IL-4/IL-13 exposure even after removal from their inflammatory environment, demonstrating that epithelial stem cells may contribute to the persistence of human disease by serving as repositories for allergic memories. This finding fundamentally changes the understanding of chronic type 2 inflammatory diseases: reduced epithelial diversity stemming from functional shifts in basal cells is a key characteristic of type 2 immune-mediated barrier tissue dysfunction, and these changes persist in progenitor cells that will give rise to future epithelial generations.

Recent studies have further characterized the molecular pathways underlying basal cell dysfunction. Two subsets of basal cells have been identified in human and murine respiratory mucosa distinguished by the expression of basal cell adhesion molecule (BCAM). BCAM expression identifies airway stem cells among the basal cell pool, and in the sinonasal mucosa, BCAM-positive basal cells expressing *TSLP*, *IL33*, *CCL26*, and the canonical basal cell transcription factor TP63 are increased in patients with chronic rhinosinusitis with nasal polyposis. IL-4/IL-13 increases the expression of *BCAM* and *TP63* through an insulin receptor substrate-dependent signaling pathway, establishing that airway epithelial remodeling in type 2 inflammation extends beyond goblet cell metaplasia to the support of a basal cell stem state poised to perpetuate inflammation (99). The potential for clinical blockade of the IL-4 receptor α-subunit to modify basal and secretory cell states *in vivo* has been demonstrated, suggesting that antagonism of downstream type 2 cytokine signaling may be able to reprogram epithelial progenitor cells and partially reverse the allergic memory stored within the basal cell compartment (98).

### 4.4 Trained immunity, remission, and early-life imprinting

As synthesized in recent reviews (100), trained immunity in type 2 immune responses extends the concept of epithelial memory beyond structural cells alone and provides a broader framework for understanding persistent airway hyperresponsiveness in asthma. In this model, prior allergen, microbial, or inflammatory exposure induces epigenetic and metabolic reprogramming in innate and barrier cell populations, resulting in exaggerated secondary responses to later stimuli. Evidence for such maladaptive memory has been described not only in airway epithelial cells, but also in macrophages and ILC2s, supporting the view that chronic asthma reflects a persistent reconfigured tissue ecosystem rather than transient inflammation alone.

Clinical remission provides particularly informative evidence for this framework. Although some patients become asymptomatic, remission is not equivalent to restoration of a healthy airway state (101). Bronchial methylation studies show that subjects in remission remain epigenetically distinct from healthy controls, indicating that prior disease can leave a persistent molecular scar. This observation may help explain why relapse remains possible despite apparent clinical improvement and why conventional measures of symptom control do not necessarily capture biological recovery.

Early life appears to be a critical window during which these long-term programs are established. Prenatal exposures, maternal immune status, and early viral infections can all shape epithelial and innate immune responses in ways that persist into later childhood or adulthood (102). Rather than acting as isolated risk factors, these exposures may function as developmental inputs that prime the airway for future disease by altering epigenetic regulation, barrier resilience, and responsiveness to subsequent insults. Taken together, trained immunity, remission-associated epigenetic persistence, and developmental imprinting support a unified explanation for the chronic, relapse-prone nature of asthma.

## 5 Part V: Therapeutic translation

The preceding analysis of epithelial memory and trained immunity reveals a fundamental constraint on current therapeutic strategies: biologics targeting downstream cytokines suppress inflammation but do not reset the epigenetic programs that sustain it.

### 5.1 Biologics targeting epithelial-immune circuits

#### 5.1.1 Current biologics and endotype-guided selection

The advent of biologic therapies has transformed the management of severe asthma, particularly for T2-high disease. Currently approved biologics include anti-IgE therapy (omalizumab), anti-IL-5/IL-5Rα therapies (depemokimab,mepolizumab, reslizumab, benralizumab), and anti-IL-4Rα therapy (dupilumab) (103). These agents have demonstrated significant clinical benefits in T2-high patients—including reductions in annualized exacerbation rates of approximately 50% (104), improvements in lung function, and reductions in oral corticosteroid use—and their endotype utility is now reflected in GINA severe asthma guidelines, which recommend biologic selection based on blood eosinophil counts, FeNO, and total IgE (103). However, these therapies have demonstrated limited or no benefit in T2-low asthma, which encompasses neutrophilic, paucigranulocytic, and obesity-associated phenotypes characterized by innate immune dysregulation, NF-κB-driven epithelial alarmin production, and steroid insensitivity. This unmet need has driven development of upstream biologics targeting epithelial alarmins, which are expressed irrespective of downstream T2 polarization and may therefore act across endotypes.

#### 5.1.2 Dupilumab: from clinical efficacy to epithelial restoration

Among downstream T2 cytokine receptor-targeting biologics, dupilumab established proof of concept that blockade of the shared IL-4/IL-13 signaling receptor can achieve both anti-inflammatory and epithelial-restorative effects in T2-high asthma. In the phase 3 QUEST trial, dupilumab reduced annualized exacerbation rates by approximately 48% overall, with the greatest benefit (∼65% reduction) in patients with baseline eosinophils ≥300 cells/μL and FeNO ≥25 ppb, confirming its optimal efficacy in the T2-high endotype (105). Building on this clinical foundation, mechanistic studies have revealed effects at the epithelial level that extend beyond inflammation suppression. When dupilumab was added to air-liquid interface cultures stimulated with IL-13, there was restoration of ciliated cell proportions in both healthy and asthmatic donors, alongside increases in ciliary beat frequency and improvements in epithelial barrier integrity. However, dupilumab shows no significant exacerbation benefit in patients with eosinophil counts below 150 cells/μL, leaving the T2-low population without an effective targeted option; and bronchial biopsy methylation data indicate that even sustained IL-4Rα blockade does not fully normalize the epigenetic signatures distinguishing asthmatic from healthy epithelium (89)—indicating that cytokine pathway blockade and epigenetic reprogramming represent complementary rather than redundant therapeutic objectives in asthma.

#### 5.1.3 Anti-alarmin therapies: evidence and limitations

Tezepelumab, a human monoclonal antibody that binds and neutralizes TSLP, represents the most advanced anti-alarmin strategy and has demonstrated efficacy across both T2-high and T2-low phenotypes. In the pivotal phase 3 NAVIGATOR trial, tezepelumab reduced the annualized asthma exacerbation rate by 56% overall; among patients with blood eosinophil counts below 300 cells/µL at baseline—a proxy for T2-low disease—the exacerbation rate was reduced by 41%, a finding that distinguishes tezepelumab from agents targeting downstream T2 cytokines (94). The phase 2 CASCADE trial further demonstrated reductions in airway submucosal eosinophils, airway hyperresponsiveness, and mucus plugging score with tezepelumab treatment (106). However, important limitations and unanswered questions remain. The mechanistic basis for tezepelumab's efficacy in T2-low disease is incompletely understood: TSLP promotes not only ILC2 and Th2 activation but also dendritic cell maturation and innate inflammatory crosstalk, yet it remains unclear which of these pathways underlies the non-eosinophilic treatment effect observed in NAVIGATOR. Furthermore, *post-hoc* analyses reveal that the magnitude of benefit in the eosinophil-low subgroup, while statistically significant, is attenuated compared to the T2-high population, and head-to-head endotype comparisons against dupilumab in T2-low disease are lacking. Whether tezepelumab modifies the underlying epithelial epigenetic landscape or simply suppresses alarmin-driven inflammation transiently remains to be established.

Anti-IL-33 therapies have also demonstrated clinical promise but present a more complex evidence landscape. Itepekimab, a human monoclonal antibody targeting IL-33 directly, significantly reduced loss-of-control events (OR vs. placebo 0.42) and improved lung function in patients with moderate-to-severe asthma not on maintenance oral corticosteroids in a phase 2b trial (107).

Importantly, itepekimab showed greater benefit in patients not receiving dupilumab, suggesting that the IL-33 and IL-4/IL-13 pathways make partially non-overlapping contributions to disease, with implications for combination or sequential biologic strategies. However, itepekimab's efficacy appears more closely linked to the T2-high subgroup: exploratory analyses in eosinophil-low patients showed attenuated or non-significant effects, raising questions about its breadth of endotype coverage compared to tezepelumab.

Tozorakimab, targeting the IL-33 receptor ST2, is in phase 2–3 development for asthma. Whether blocking ST2 confers advantages over direct IL-33 neutralization—particularly in T2-low disease where IL-33 contributes to innate barrier disruption—remains to be established in adequately powered endotype-stratified trials.

Astegolimab, a ST2-targeting antibody evaluated in the phase 2 ZENYATTA trial, reduced the annualized exacerbation rate by up to 43% in a broad population of severe asthma patients; notably, AER reductions in eosinophil-low patients were comparable to those in the overall population (54% at the 490-mg dose), providing proof-of-concept support for the anti-IL-33/ST2 axis as a pan-endotype target (108), though the magnitude of benefit in T2-low subgroups was again more modest than in eosinophilic disease.

### 5.2 Epigenetic and RNA-based therapies

#### 5.2.1 HDAC inhibitors: endotype relevance and translational challenges

Histone deacetylase inhibitors represent a mechanistically attractive therapeutic class with distinct rationale across endotypes. In T2-high disease, elevated HDAC1/9, SIRT6, and SIRT7 expression driven by IL-4/IL-13 represses tight junction gene transcription; HDAC inhibition restores barrier integrity to levels comparable to healthy controls, complementing rather than duplicating anti-IL-13 biologic action (109). In T2-low and steroid-insensitive disease, HDAC2 expression is specifically reduced, impairing glucocorticoid receptor deacetylation and corticosteroid responsiveness; low-concentration theophylline partially restores HDAC2 activity in neutrophilic models (90), highlighting a T2-low-specific therapeutic opportunity where conventional biologics are least effective.

In murine allergic airway models, selective HDAC6 inhibition (Tubastatin A), HDAC8 inhibition (PCI-34051), and broad-spectrum inhibition (givinostat) all reduced airway inflammation, remodeling, and hyperresponsiveness (110). However, systemic tolerability concerns—immunosuppression and cytopenias—argue against pan-HDAC inhibition for a non-life-threatening chronic disease. Inhaled delivery and isoform-selective strategies targeting HDAC1/9 (T2-high) or HDAC2 restoration (T2-low) are priority approaches to improve the therapeutic index (110). DNMT inhibitors (low-dose azacitidine) and EZH2 inhibitors have shown preclinical promise in reducing trained immunity-associated hyperresponsiveness (62), but endotype-specific efficacy and airway safety require systematic evaluation.

#### 5.2.2 MicroRNA-based therapeutics: endotype-stratified opportunities

In T2-high disease, miR-21 (upregulated by IL-13, suppresses *PTEN/IL12A*), miR-145 (amplifies TGF-β/SMAD3 signaling), and miR-155 (suppresses *SOCS1/SHIP1* to amplify type 2 signaling) are established therapeutic targets; antagomir-mediated inhibition of each reduces eosinophilic inflammation, MUC5AC expression, and partially restores steroid sensitivity in murine models. In T2-low disease, miR-223-3p regulates NLRP3 inflammasome activity and IL-1β production, while miR-363-3p (induced by IL-17A/TNF-α) suppresses barrier components *CLDN8*, *PCDH1*, and *PTEN*—both representing endotype-specific targets. Let-7 family members, downregulated across severe asthma, target *IL13*, *IL33*, and *TSLP* mRNAs; their restoration suppresses alarmin production across endotypes (111). The lncRNA *PVT1*, elevated specifically in corticosteroid-insensitive severe asthma, may represent a T2-low-relevant target for airway remodeling.

Delivery platforms using LNA anti-miRs and inhaled lipid nanoparticles have demonstrated feasibility and acceptable tolerability in murine airway models. Endotype-matched patient selection via bronchial epithelial miRNA profiling will be essential for informative early-phase clinical trials.

### 5.3 Biomarkers, remission, and precision medicine

Blood eosinophils, FeNO, and total IgE have established value for T2-high biologic selection, while periostin shows promise as a serum surrogate of epithelial T2 activation. These markers are substantially less informative in T2-low disease, where no validated biomarker currently guides therapeutic selection.

Emerging transcriptomic and epigenetic approaches offer a more mechanism-based framework. In T2-high disease, the three-gene epithelial T2 signature (*CLCA1, POSTN, SERPINB2*) stratifies patients with greater endotype granularity than blood biomarkers alone (92). For T2-low disease, lower bronchial epithelial CD8⁺ T-cell network expression and elevated plasma IL-6 show promise as molecular discriminators, though neither has been prospectively validated (88). Epithelial DNA methylation at loci associated with *IL4, TSLP*, and tight junction genes may provide stable, cell-intrinsic measures of epigenetic disease burden that persist across clinical states (112); circulating and bronchoalveolar miRNA profiles (miR-21/let-7 in T2-high; miR-223-3p/miR-363-3p in T2-low) show endotype-associated patterns in discovery cohorts pending prospective validation.

Clinical remission—defined by T2-BREATHE/GINA consensus as sustained absence of exacerbations, symptom control, and preserved lung function for ≥12 months—is an increasingly recognized therapeutic target. However, symptom-level remission does not equate to biological recovery: bronchial biopsy methylation studies demonstrate that remission patients remain epigenetically distinct from healthy controls at barrier-function and T2-activation loci (89), indicating persistence of a molecular disease scar that may underlie residual relapse risk. The ultimate goal of precision medicine in asthma must therefore extend beyond inflammation suppression to encompass functional barrier restoration and, ultimately, epigenetic normalization—tracked through epithelial methylation remodeling, tight junction gene expression, and alarmin secretion capacity rather than inflammatory control alone.

Translating these candidate markers into validated clinical tools requires a structured progression from discovery through qualification to validation—a challenge that intersects directly with the trial design priorities as discussed in Section 5.4 below.

### 5.4 Unmet needs and future directions

Important unmet needs persist across both endotypes. In T2-high disease, approximately 40%–50% of patients on approved biologics fail to achieve clinical remission, and post-treatment epigenetic analyses confirm that no current agent fully resets the molecular programs of chronic disease. In T2-low asthma, no approved biologic is specifically indicated, and mechanistic heterogeneity within this endotype—neutrophilic, paucigranulocytic, obesity-associated, corticosteroid-insensitive—complicates trial design and biomarker development.

Four priorities follow from these gaps: ① defining which epithelial epigenetic changes are causally linked to disease persistence, stable across clinical states, and pharmacologically reversible (62); ② integrating multi-omics (methylation, histone modification, single-cell transcriptomics, spatial resolution) to map actionable epithelial endotypes for biomarker-guided trials (112); ③ developing inhaled, isoform-selective epigenetic agents alongside companion diagnostics matched to specific dysregulation patterns (110); ④ mechanistically dissecting the endotype-specific basis for anti-alarmin efficacy in T2-low disease to enable rational combination strategies (94).

Advancing the field requires structured biomarker validation that proceeds through discovery, qualification, and formal validation phases. Qualification is most efficiently achieved by embedding biomarker substudies with pre-specified outcome hypotheses within ongoing biologic trials—for example, within the PRECISE adaptive platform (113)—rather than conducting standalone observational studies with insufficient statistical power. Nasal epithelial brushings offer a minimally invasive and pediatric-feasible surrogate for bronchial sampling, given well-established correlations between nasal and bronchial epithelial gene-expression and methylation profiles; systematic validation of nasal-based biomarker performance against bronchoscopic reference standards constitutes a near-term priority. Conventional trial endpoints (AER, FEV₁, symptom scores) are likely insensitive to the upstream molecular changes that epigenetic therapies are designed to correct; more informative surrogates—bronchial biopsy tight junction immunostaining (claudin-18, ZO-1), epithelial transcriptomic profiling, and DNA methylation at sentinel IL-13-associated CpG loci—should therefore be incorporated as pre-specified secondary endpoints in early-phase trials. Adaptive designs with interim biomarker analyses are better suited to asthma's endotype heterogeneity than traditional fixed-dose parallel-group designs (113), and regulatory approval of novel epigenetic agents will require demonstration of durable benefit beyond the active treatment period.

**Near-term priorities** (clinically actionable): refining endotype-guided biologic selection using validated biomarkers (113); advancing tezepelumab's use in T2-low disease through mechanistic substudies; and qualifying epithelial biomarkers—claudin-18 expression, tight junction gene methylation, miRNA endotype profiles—as trial-ready endpoints.

**Longer-term priorities** (requiring foundational work): inhaled HDAC inhibitors must demonstrate isoform selectivity and pulmonary tolerability; anti-miR strategies require validated delivery platforms with cell-type-specific uptake in basal progenitor cells; CRISPR-based epigenetic editing remains early preclinical.

## 6 Part VI: Conclusion

This review supports a shift from a predominantly T-cell-centered view of asthma toward a model in which the airway epithelium functions as a primary site of disease initiation, amplification, and persistence. Barrier disruption, mucus dysfunction, alarmin release, and altered epithelial cell states do not simply accompany inflammation; they help organize it. Genetic susceptibility and environmental exposure converge on the epithelium, while epigenetic mechanisms provide the molecular means by which transient insults become durable pathological programs.

Within this framework, epithelial memory and trained immunity offer a compelling explanation for several unresolved features of asthma, including chronicity, relapse after apparent remission, and incomplete responses to conventional anti-inflammatory therapy. The ability of epithelial progenitors and innate immune cells to retain maladaptive transcriptional and epigenetic states suggests that long-term disease control may require more than suppression of downstream cytokines. It may also require restoration of barrier competence and partial rewiring of pathological cellular memory.

These insights have important translational implications. Upstream biologics targeting epithelial-immune signaling, along with emerging epigenetic and RNA-based therapies, point toward a future in which treatment is guided not only by inflammatory biomarkers but also by epithelial and molecular endotypes. A major next step for the field will be to define robust biomarkers of epithelial dysfunction and memory, clarify which changes are reversible, and determine whether intervention during early or preclinical stages can alter long-term disease trajectories. Ultimately, a more complete understanding of airway epithelial dysfunction and epigenetic memory may help move asthma care beyond symptom control toward durable remission and true disease modification.

## Abbreviations

AER: annualized exacerbation rate
AHRR: aryl hydrocarbon receptor repressor
ALI: air-liquid interface
BCAM: basal cell adhesion molecule
BMI: body mass index
CBP: CREB-binding protein
CCL17: C-C motif chemokine ligand 17
CDHR3: cadherin-related family member 3
CLCA1: chloride channel accessory 1
CLDN: claudin
COPD: chronic obstructive pulmonary disease
CpG: cytosine-phosphate-guanine
CRISPR: clustered regularly interspaced short palindromic repeats
CXCL: C-X-C motif chemokine ligand
DC: dendritic cell
DNMT: DNA methyltransferase
EGFR: epidermal growth factor receptor
ERK: extracellular signal-regulated kinase
EWAS: epigenome-wide association study
EZH2: enhancer of zeste homolog 2
FAM13A: family with sequence similarity 13 member A
FeNO: fractional exhaled nitric oxide
FEV₁: forced expiratory volume in 1s
FLG: filaggrin
GINA: global initiative for asthma
GSDMB: gasdermin B
GWAS: genome-wide association study
HAT: histone acetyltransferase
HDAC: histone deacetylase
H3K9me3: histone H3 lysine 9 trimethylation
H3K18ac: histone H3 lysine 18 acetylation
ICS: inhaled corticosteroid
IgE: immunoglobulin E
IL: interleukin
IL-4Rα: interleukin-4 receptor alpha subunit
IL-17RB: interleukin-17 receptor B
ILC2: type 2 innate lymphoid cell
KCNH2: potassium voltage-gated channel subfamily H member 2
lncRNA: long non-coding RNA
LNA: locked nucleic acid
MALAT1: metastasis-associated lung adenocarcinoma transcript 1
MAPK: mitogen-activated protein kinase
MEK: mitogen-activated protein kinase kinase
miRNA: MicroRNA
MUC5AC: mucin 5AC
MUC5B: mucin 5B
ncRNA: non-coding RNA
NF-κB: nuclear factor kappa B
NLRP3: NLR family pyrin domain-containing 3
NOTCH4: notch receptor 4
OR: odds ratio
ORMDL3: ORMDL sphingolipid biosynthesis regulator 3
PCDH1: protocadherin 1
PI3K: phosphoinositide 3-kinase
PM2.5: particulate matter with aerodynamic diameter ≤2.5 μm
POSTN: periostin
PTEN: phosphatase and tensin homolog
PVT1: plasmacytoma variant translocation 1 lncRNA
ROS: reactive oxygen species; SERPINB2, serine protease inhibitor B2
SIRT: sirtuin
SMAD3: SMAD family member 3
SNHG16: small nucleolar RNA host gene 16
SOCS1: suppressor of cytokine signaling 1
SPDEF: SAM pointed domain-containing ETS transcription factor
ST2: suppression of tumorigenicity 2 (IL-33 receptor)
STAT6: signal transducer and activator of transcription 6
T2-high: type 2-high (eosinophilic) asthma endotype
T2-low: type 2-low asthma endotype
TARC: thymus and activation-regulated chemokine
TGF-β: transforming growth factor beta
Th2: T helper 2 cell
TJ: tight junction
TNF-α: tumor necrosis factor alpha
TP63: tumor protein p63
TSLP: thymic stromal lymphopoietin
TSLPR: thymic stromal lymphopoietin receptor
XIST: X-inactive specific transcript
ZO-1: zonula occludens-1
